# Innate immunity and immunotherapy for hemorrhagic shock

**DOI:** 10.3389/fimmu.2022.918380

**Published:** 2022-08-25

**Authors:** Qingxia Huang, Song Gao, Yao Yao, Yisa Wang, Jing Li, Jinjin Chen, Chen guo, Daqing Zhao, Xiangyan Li

**Affiliations:** ^1^ Research Center of Traditional Chinese Medicine, College of Traditional Chinese Medicine, Changchun University of Chinese Medicine, Changchun, China; ^2^ Jilin Ginseng Academy, Key Laboratory of Active Substances and Biological Mechanisms of Ginseng Efficacy, Ministry of Education, Jilin Provincial Key Laboratory of Bio-Macromolecules of Chinese Medicine, Changchun University of Chinese Medicine, Changchun, China; ^3^ Jilin Xiuzheng Pharmaceutical New Drug Development Co., Ltd., Changchun, China

**Keywords:** Innate immunity, hemorrhagic shock, immunotherapy, multiple organ failure, mesenchymal stem cell, antibody therapy, small molecule inhibitor

## Abstract

Hemorrhagic shock (HS) is a shock result of hypovolemic injury, in which the innate immune response plays a central role in the pathophysiology ofthe severe complications and organ injury in surviving patients. During the development of HS, innate immunity acts as the first line of defense, mediating a rapid response to pathogens or danger signals through pattern recognition receptors. The early and exaggerated activation of innate immunity, which is widespread in patients with HS, results in systemic inflammation, cytokine storm, and excessive activation of complement factors and innate immune cells, comprised of type II innate lymphoid cells, CD4^+^ T cells, natural killer cells, eosinophils, basophils, macrophages, neutrophils, and dendritic cells. Recently, compelling evidence focusing on the innate immune regulation in preclinical and clinical studies promises new treatment avenues to reverse or minimize HS-induced tissue injury, organ dysfunction, and ultimately mortality. In this review, we first discuss the innate immune response involved in HS injury, and then systematically detail the cutting-edge therapeutic strategies in the past decade regarding the innate immune regulation in this field; these strategies include the use of mesenchymal stem cells, exosomes, genetic approaches, antibody therapy, small molecule inhibitors, natural medicine, mesenteric lymph drainage, vagus nerve stimulation, hormones, glycoproteins, and others. We also reviewed the available clinical studies on immune regulation for treating HS and assessed the potential of immune regulation concerning a translation from basic research to clinical practice. Combining therapeutic strategies with an improved understanding of how the innate immune system responds to HS could help to identify and develop targeted therapeutic modalities that mitigate severe organ dysfunction, improve patient outcomes, and reduce mortality due to HS injury.

## Introduction

Hemorrhagic shock (HS) is a life-threatening condition occurring in various clinical situations, including trauma, childbirth, gastrointestinal hemorrhage, and aneurysmal rupture (1, 2). It is a represents a substantial global problem, which more than 1.9 million deaths per year worldwide, of which 1.5 million results from traumatic injury (3). Although 20% of the fatal cases of HS are considered avoidable, current practice has failed to improve the survival rate (4). Clinically, HS is treated with an expedited anatomic control of bleeding in conjunction with intravenous blood products, deliberate hypotension, antifibrinolytic therapy, and vasodilation (5, 6). Despite advances in clinical treatment aimed at the hypovolemic injury, patients who survive the initial HS insult have severe complications due to organ reperfusion injury, delayed infections, immune dysfunction, and the risk of developing organ, with incidences of 37.2%, or multiple organ failure (MOF), with incidences of 22.1% (7, 8).

Increasing evidence has proved that the modulation of innate immune responses is a promising therapeutic strategy for preventing and treating HS-induced MOF injury and complications (9). Soon after HS insult, exposure to exogenous pathogen-associated molecular pattern molecules (PAMPs) and endogenous damaged-associated molecular pattern molecules (DAMPs) extensively activate the innate immune defense, mainly comprising type II innate lymphoid cells (ILC2), CD4^+^ T cells, natural killer (NK) cells, eosinophils, dendritic cells (DCs), basophils, macrophages, neutrophils, and the complement cascade (10). The innate immune response initiated and propagated in response to HS triggers inflammatory and anti-inflammatory mechanisms within 30 min post-injury, followed by systemic immune response syndrome (SIRS) and counterbalancing anti-inflammatory response syndrome (CARS), which are related to organ injury and complications (11). The systemic parameters during HS suggest that restoration of innate immunity offers exciting and promising directions for developing novel therapeutics for HS-induced second injuries. Recently, compelling evidence has focused on the innate immune response for the monitoring and therapy of HS (8, 9, 12, 13). The emerging understanding is poised to revolutionize the treatment of HS through targeted immune modulators.

In this article, we firstly summarize recent advances in the pathomechanistic insights associated with the innate immune response following HS injury, then systematically detail the cutting-edge therapeutic strategies used in the past decade regarding the innate immune regulation in this field, such as mesenchymal stem cells (MSCs), MSC-derived exosomes, MSC-derived extracellular vesicles (MSC-EVs), MSC-derived soluble factors (FS-MSC), genetic approaches, antibody therapy, small molecule inhibitors, natural medicine, mesenteric lymph (ML) drainage, vagus nerve stimulation (VNS), hormones, glycoproteins, and others. We also reviewed clinical studies on the regulation of immunity for treating HS and assessed the potential of immune regulation concerning a translation from basic research to clinical practice. Combining these therapeutic strategies with an improved understanding of how the innate immune system responds to HS could help to identify and develop targeted therapeutic modalities that mitigate severe organ dysfunction, improve patient outcomes, and reduce mortality due to HS injury.

## Innate immune response to HS

**Figure 1:**
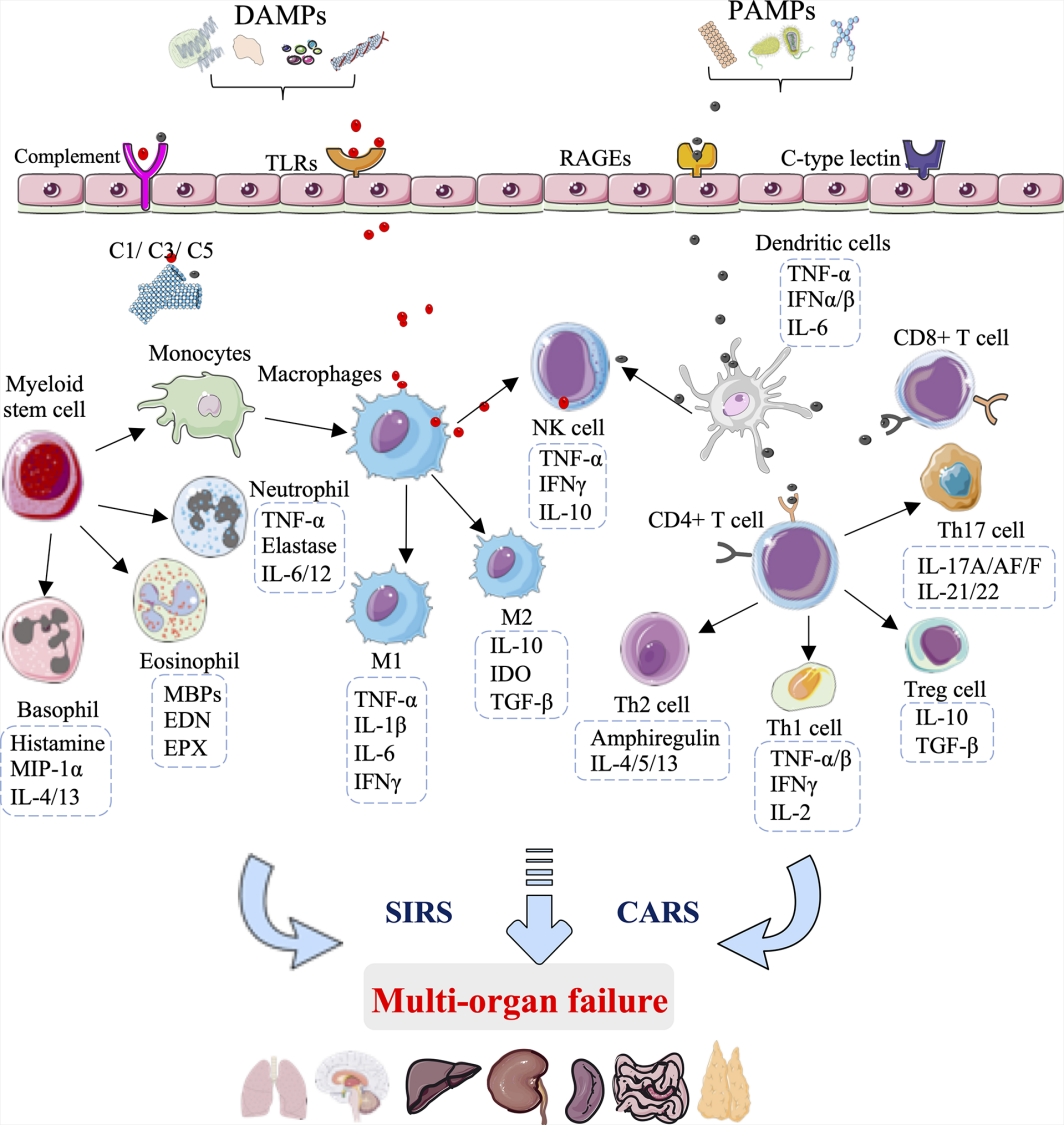
Innate immunity-mediated pathomechanisms in multi-organ failure development after hemorrhagic shock. During the development of hemorrhagic shock, the innate immunity rapid response to damage-associated molecular patterns (DAMPs) and pathogen-associated molecular patterns (PAMPs) is mediated through pattern recognition receptors, including toll-like receptors (TLRs), receptors of advanced glycation end products (RAGEs), C-type lectin receptors, and complement receptors. The early and exaggerated activation of innate immunity results in excessive activation of complement factors(C1/C3/C5) and innate immune cells, comprising macrophages, DC cells, T cells, natural killer (NK) cells, eosinophils, basophils, and neutrophils. The activation of innate immune cells leads to the secretion of cytokines and chemokines, which exaggerates inflammation and subsequent immunosuppression, causing systemic immune response syndrome (SIRS) and a counterbalancing anti-inflammatory response syndrome (CARS), ultimately leading to multi-organ failure.

Increasing experimental and clinical evidence indicates that innate immunity is the predominant mediator in the pathophysiology of HS injury that unequivocally leads to organ damage and failure. The innate immune activation and immunosuppression responses to HS injury obtained in clinical trials and preclinical experiments are summarized in **Figure 1**.

At the site of hemorrhage, the immune system is challenged with “alarmins”, among which exogenous PAMPs are expressed on invading microorganisms and endogenous DAMPs are released from damaged and host cells, and include mitochondrial DNA, cold-inducible RNA-binding protein (CIRP), high mobility group box 1 (HMGB1), interleukin (IL)-25, IL-33, mitochondrial N-formyl peptides (F-MIT), and F-Actin (14–16). These “alarmins” are recognized by distressed immune cells through groups of pattern-recognition receptors, including toll-like receptors (TLRs), receptors of advanced glycation end products (RAGEs), C-type lectin receptors, and complement receptors (17). The excessive proinflammatory response SIRS and parallel immunosuppression CARS are induced after these damage molecules engage with their corresponding receptors, which is characterized by the release of cytokines, chemokines, complement factors, and coagulation proteins, as well as activation of innate immune cells (18). In terms of negative feedback, the excessive innate immune response can promote the circulation of new DAMPs, thereby amplifying a vicious cycle of cell and tissue injuries (19, 20).

During the development of HS, the innate immune cells act as the first line of defense, providing a rapid response to pathogens or danger signals through pattern recognition receptors (21). One of the first innate immune cell types to the site of injury is antigen-presenting cells, including tissue-resident macrophages, which sense damaging molecules and then differentiate from M1-type macrophages that secrete proinflammatory factors (TNF-α, IL-1β, IL-6, and interferon-γ) to M2-type macrophages that secrete anti-inflammatory factors (IL-10, IGF-1, and TGF-β) (22). In addition to defending against pathogens, macrophages are crucial to the maintenance of tissue homeostasis (23). Indeed, data suggest that the macrophage phenotype can correspondingly switch from M1-type macrophages, polarized by Th1 cytokines (GM-CSF, TNF-α, and interferon-γ), to M2-type macrophages, polarized by Th2 cytokines (IL-4 and IL-13) to deal with tissue repair (24, 25). Interestingly, neutrophils also regulate T cell function, and the M1-to-M2 macrophage switch represents a central element in the clearance of neutrophils by efferocytosis (26–28).

DCs are another type of antigen-presenting cell, which deliver antigens to T and NK cells. DCs show rapid responsiveness to pathogens or danger signals, which is followed by the secretion of TNF-α, interferon (IFN)-α, IFN-β, as well as IL-6 within a few hours after HS (29). The activation of T lymphocytes by DCs or danger signals is essential in exaggerating inflammation and subsequent immunosuppression (30). CD4^+^ T cells are the primary lymphocytes involved in HS injury and are classically divided into four categories: Th1, Th2, Th17, and T regulatory (Treg) cells (31). Treg cells can suppress T-cell activation and Th1 cytokine production after injury. Data suggest that HMGB1 binding to RAGE, TLR4 and TLR9 can promote the activation of DCs, CD4^+^ T, CD8^+^ T, Th17, and Treg cells in response to HS injury (32–34). The imbalance of Th17/Treg has been suggested to be positively correlated with the degree of acute liver injury (35). The balance of Th1/Th2 is attributed to conversion to type 2 responses during HS development (36–38). DCs subject to HS were more inclined to polarize naive CD4^+^ T cells into Th2 and Treg cells, consistent with the clinically observed immunosuppressive phenomena in severe patients (39). Clinically, the peak of organ damage and MOF occurs within the 3 days of HS, with lung failure being the most common (40, 41).

Taken together, innate immunity is activated early after HS injury, and cross-talk between various immune cells ultimately results in MOF. Many attempts of therapeutic strategies focusing on the innate immune regulation in preclinical and clinical studies have achieved promising results in reducing tissue injury, organ dysfunction, and ultimately mortality due to HS.

## New insights into HS therapeutics with innate immune regulation

### Stem cell therapy in HS

Stem cells possess a remarkable potential for developing new cell-based treatments in the context of HS by modulating local and systemic deleterious immune responses (42, 43). **Table 1** shows the main stem cell-related therapeutic strategies in the HS model in order of stem-cell type: MSC (MSCs, MSC-EVs, FS-MSC, MSC-derived exosomes, and IL-1β primed MSC), adipose-derived stem cells, and neutrophil progenitors. Each of these therapeutic strategies is discussed in detail below.

**Table 1:** Overview of the applications of stem cell-related therapeutic strategies in a hemorrhagic shock model.

Treatment strategy	HS model	Mechanism	Inhibited outcome	Refs
MSC	Rat	Increasing Treg cell population in the peripheral blood	Lung injury	(44, 45)
MSC	Rat	Decreasing leukocytes (CD68^+^ and MPO^+^ cells) infiltrates	Lung injury	(46)
MSC or MSC-EVs	Mice	Reducing the level of inflammatory -chemokine and cytokines in the lungs	Lung injury	(47)
MSC-EVs	Porcine	Downregulating the inflammation -related transcription in the brain	Brain injury	(48)
MSC-EVs	Mice	Mediating M2-type macrophage polarization and immunosuppression	Liver injury	(49)
FS-MSC	Rat	Reducing inflammation and neutrophil infiltration in the lung	Lung injury	(50)
MSC-derived exosomes	Swine	Decreasing the secretion of IL-1, IL-6, IL-18, and increasing GMC-SF levels	Neurologic injury	(51, 52)
IL-1β primed MSC	Rat	Decreasing systemic cytokines (IL-1α, IL-6, and IL-10) and the PD-1/PD-L1 axis	MOF injury	(53)
Adipose-derived stem cells	Rat	Inhibiting IL-6 secretion in plasma	Liver injury	(54)
Neutrophil progenitors	Mice	Decreasing proinflammatory cytokines and increasing neutrophil migration into the airspace	Lung infection	(55)

MSCs are multipotent stem cells, which are commonly used as a clinical cell therapeutic strategy for immunomodulation and tissue repair (9, 56). Treg cells, an immunosuppressive T cell subset, are essential for maintaining immune homeostasis and tolerance (57). In a rat model of unilateral lung contusion followed by HS, impaired wound healing and lung structure were improved by MSCs treatment by increasing the Treg cell population (44, 45). Cell-based therapies using MSCs or MSC-EVs are beneficial for improving neurologic outcomes and lung injury in animal models of HS (9). Moreover, in an HS-induced mild lung injury rat model, leukocyte infiltrates (CD68^+^ and MPO^+^ cells) were significantly reduced in the lung after treatment with MSCs (46). The latest transcriptome data demonstrated that treatment with MSCs or MSC-EVs was associated with the inactivation of inflammation-chemokine and cytokine pathways in the lung of HS mice (47). In a porcine model of HS, the neuroprotective and neurorestorative properties observed in MSC-EVs treatment were also associated with the attenuation of inflammation-related transcription in the brain (48).

A previous study showed that IL-10, an immunoregulatory cytokine, binds to the IL-10 receptor and inhibits inflammation following HS (58, 59). Yunwei Zhang et al. found that IL-10-deficient MSCs lost the protective function compared to WT MSCs in an HS-induced hepatic injury model (49). Another experiment indicated that MSC-EVs carrying IL-10 as cargo were mainly taken up by macrophages in the liver, mediating M2-type macrophage polarization and consequent immunosuppression in HS-induced hepatic injury (49). Neutrophils are the first innate immune cells against pathogens due to their array of microbicidal activities (60, 61). Clinically, the drop in circulating neutrophils is positively correlated with the occurrence of MOF (62, 63). FS-MSCs have an immunomodulatory action through paracrine activity by secreting anti-inflammatory cytokines and growth factors (64). Recent studies have shown that treatment with FS-MSCs significantly reduced inflammation and lung neutrophil infiltrates in an HS-induced rat model (50).

An early single dose of exosomes derived from MSC treatment has been shown to attenuate neurological injury by decreasing IL-1, IL-6, and IL-18, and increasing granulocyte-macrophage colony-stimulating factor (GM-CSF) levels in the Yorkshire swine model of HS (51). Additionally, the administration of human MSC-derived exosomes induces transcriptomic changes of neuroinflammation after HS injury in swine (52). In several *in vitro* and *in vivo* studies, IL-1β priming maximized the immunomodulation effect of MSCs by regulating IL-6 and IL-8 expression and influencing the polarization of peritoneal macrophages (65, 66). Moreover, systemic cytokines (IL-1α, IL-6, and IL-10) and the programmed cell death receptor (PD)-1/PD-L1 axis were decreased by IL-1β-primed MSCs on monocytes and granulocytes in HS-induced kidney and liver injury model (53). Similarly, the IL-6 concentration also decreased with adipose-derived stem cell treatment in HS-induced liver injury (54). In rodent models of HS, it has been demonstrated that, in addition to suppressive proinflammatory cytokines in the lungs, there is an increase in neutrophil migration into the airspace from the bone marrow after neutrophil progenitor transplant, which can be used in the treatment and prevention of secondary infection following HS (55).

**Figure 2:**
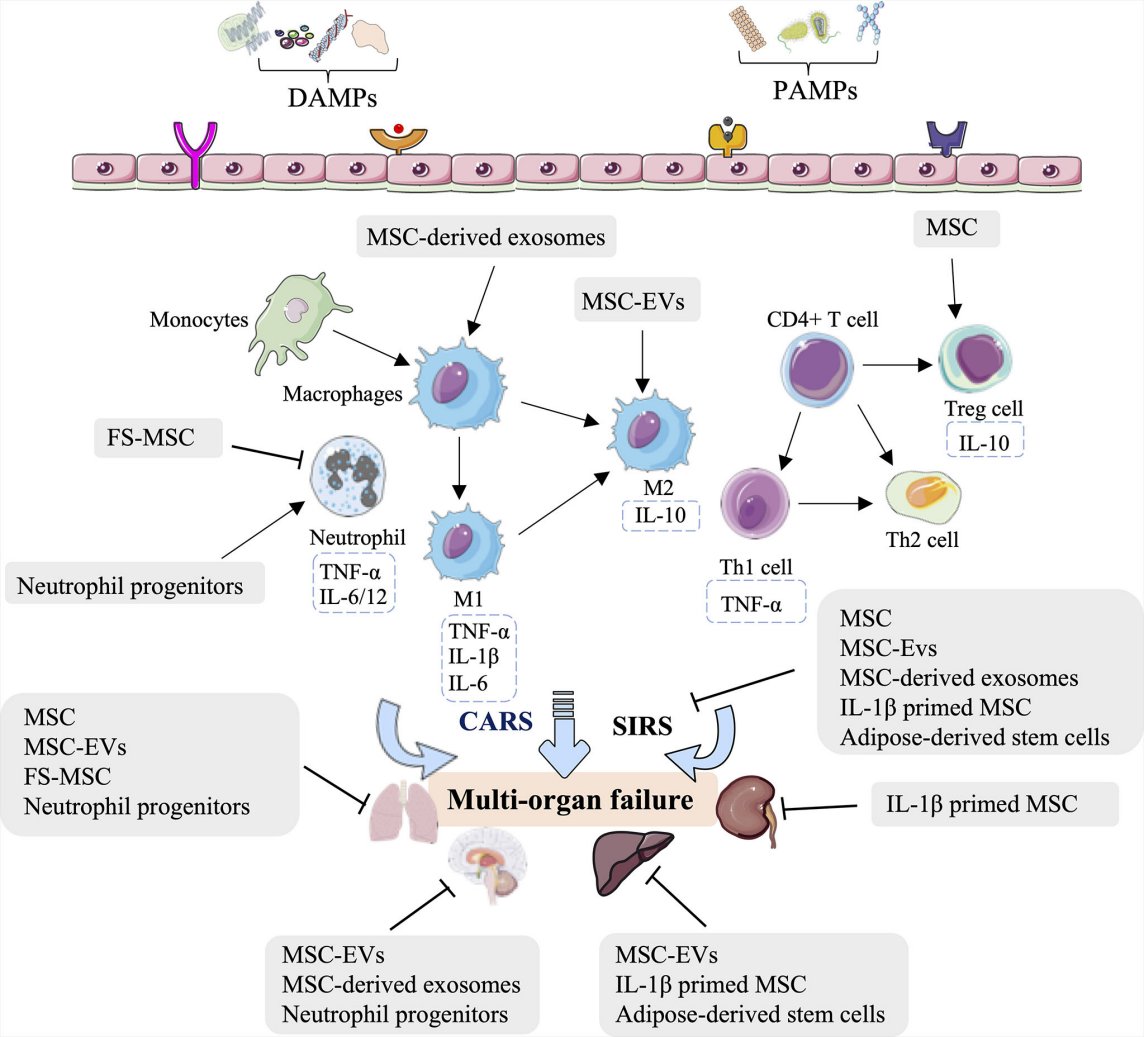
Overview of the immune therapeutic strategies of stem cells in hemorrhagic shock. The mesenchymal stem cell (MSC), MSC-derived extracellular vesicles (MSC-EVs), MSC-derived soluble factors (FS-MSC), MSC-derived exosomes, adipose-derived stem cells, and neutrophil progenitors can relieve the lung, neurologic, kidney, and liver injury by regulating neutrophil infiltration, increasing the Treg population, mediating M2-type macrophage polarization and consequent immunosuppression, and inactivation of inflammatory chemokine and cytokines.

Indeed, several studies—mostly in rodents but also in porcine—have concluded that MSCs, MSC-EVs, FS-MSCs, MSC-derived exosomes, IL-1β-primed MSCs, adipose-derived stem cells, and neutrophil progenitors can relieve lung, neurologic, kidney, and liver injury by regulating innate immunity (**Figure 2**, **Table 1**). The innate immune processes involved in the above effects include reducing the leukocyte and neutrophil infiltrate, increasing the Treg population, mediating M2-type macrophage polarization and consequent immunosuppression, and inactivating inflammatory chemokines and cytokines (**Figure 2**, **Table 1**). These stem cell-related therapeutic strategies represent a potential opportunity for treating HS-induced second injuries.

### Antibody therapy and genetic approaches in HS

IL-6 plays a prominent role in the differentiation from Th1 to Th2 in the development of HS (67). The secretion of IL-6 is positively correlated with the prognosis patients with shock and organ dysfunction (68, 69). Zhang Yong et al. reported that treatment with anti-mouse IL-6 monoclonal antibody immediately before resuscitation can prevent Th2 cytokine production, suppress the lymphocyte response, reduce the level of IL-10, keratinocyte-derived chemokine (KDC), monocyte chemoattractant protein 1(MCP-1), and macrophage inhibitory protein 1 (MIP-1) in a mouse model combining HS and lower-extremity injury (67). The mucosal address in cell adhesion molecule-1 (MAdCAM-1), a critical mediator of the early innate immune response to HS, mainly mediates lymphocyte recruitment to the gut during the inflammatory storm phase (70–72). This observation is supported by a finding that antibody blockade of MAdCAM-1 can decrease the secretion of IL-1β, IL-6, and TNF-α, reduce lymphocyte infiltration, ameliorate intestinal barrier dysfunction, and prolong survival (70). B and T lymphocyte attenuator (BTLA), a receptor that is structurally similar to PD-1, is expressed on T lymphocytes, B lymphocytes, monocytes, macrophages, and DCs (73, 74). A previous study showed that treatment with the anti-BTLA monoclonal [6A6] antibody (25μg/g body weight) can abolish HS followed by sepsis-induced reduction of cytokines and chemokines (TNF-α, IL-12, IL-10, KC, MIP-2, MCP-1) and decreased recruitment of neutrophils, macrophages, and DCs to the peritoneal cavity, which in turn relieve organ injury and reduce mortality (75).

Clinical studies have shown patients with HS with a poor prognosis within 24 h after admission have higher type 2 cytokines in serum, such as IL-5 (76). IL-33-stimulated ILC2, the resident innate lymphocytes that potently regulate host immunity in the lung, are the primary source of type 2 cytokines response to HS injury (34, 77). A recent finding has shown that anti-IL-5 antibody, IL-33, or ILC2 deletion, significantly increased IL-5 expression in neutrophils and decreased lung injury scores at 6 h in the HS-induced mice injury model (77). DAMPs can activate systemic inflammation and organ injury in HS through binding to TLR2 on immune cells (78). Similarly, anti-TLR2 monoclonal antibody or TLR2^-/-^ mice exhibited significantly less liver damage, and lower NF-κB and inflammatory cell infiltrate in HS at 20 h (78). Consistently, the phenotype of TLR2^-/-^ mice shows reduced intestinal injury accompanied by reduced complement (CD55, Factor H, and C3) and inflammatory (IL-12, IL-6, and TNF-α) factors, compared to wild-type mice (79).

Extracellular CIRP, an 18-kDa RNA chaperone protein, acts as an endogenous proinflammatory mediator, binds to TLR4, and leads to mitochondrial DNA fragmentation that triggers innate immunity and inflammatory responses in patients with HS (17, 80). Continuity studies demonstrated that the purified recombinant murine CIRP (rmCIRP) induces cytokine release in macrophages and deficiency or blockade of CIRP using antisera leads to attenuated TNF-α and IL-6 release, neutrophil accumulation, and lethality in HS injury (17, 80). Interestingly, wound-associated TNF-α enhancement and neutrophil infiltration is also attenuated in CIRP^-/-^ mice compared to WT mice (81). Mitochondrial DNA binds to the stimulator of interferon genes (STING) as a ligand, activating ype I interferon and proinflammatory cytokines-producing signals (82, 83). Kehong Chen et al. reported that the HS-induced increase in IL-6 and IFN-β levels in the serum and the high mRNAs expression of TNF-α, IL-6, and IL-1β in the lung were significantly counteracted by STING knockout, which suggests that the absence of STING significantly reduces inflammation and lung injury after HS (84).

Many studies have focused on the role of PD-1 and its ligand, PD-L1 (B7H1) in the cellular immunotherapy (85–87). The population of PD-1^+^ blood leukocytes in patients is positively correlated with interleukin levels in the serum, which suggests that PD-1 is a key indicator in the assessment of HS-induced immune dysfunction (87). Indeed, in terms of immune regulation, animals deficient in PD-1 or PD-L1 expression exhibited an attenuation in the neutrophil influx in HS injury, while PD-L1 knockout produced a marked suppression in the secretion of TNF-α, IL-6, and MCP-1, which were consistently elevated induced by HS in the WT mice group (88).

Clinical and preclinical studies have observed that nuclear factor-erythroid 2 p45-related factor-2 (Nrf2), a major mediator in innate immunity and inflammation, is significantly increased in the leukocytes collected from patients with HS (89–92). Haige Zhao et al. reported that HS-induced secretion of HMGB1, L-6, IL-1β, and TNF-αwere higher at 2 h in Nrf2 knockout mice (92). Likewise, Nrf2-KO offers no benefit over the hepatoprotection of remote ischemic conditioning in reductions in HS-induced TNF-α and IL-6 (93).

CD226, a costimulatory adhesion molecule expressed on both immune and endothelial cells, can regulate immune metabolic activity and function (94, 95). Recent studies have illustrated that CD226 deficiency in vascular endothelial cells can alleviate HS-induced intestinal damage and the inflammatory response (96). Emerging evidence shows that microRNAs play essential roles in pathophysiological responses by regulating inflammation and immunity (97, 98). Moreover, data suggest that miR-18b-5p knockdown notably reduced the levels of SOD1, iNOS, and IL-6 in macrophages, decreased the M1/M2 ratio of macrophages, and reduced the Th1/Th2 ratio of CD4^+^ T cells in splenic tissues after HS injury (99).

**Figure 3:**
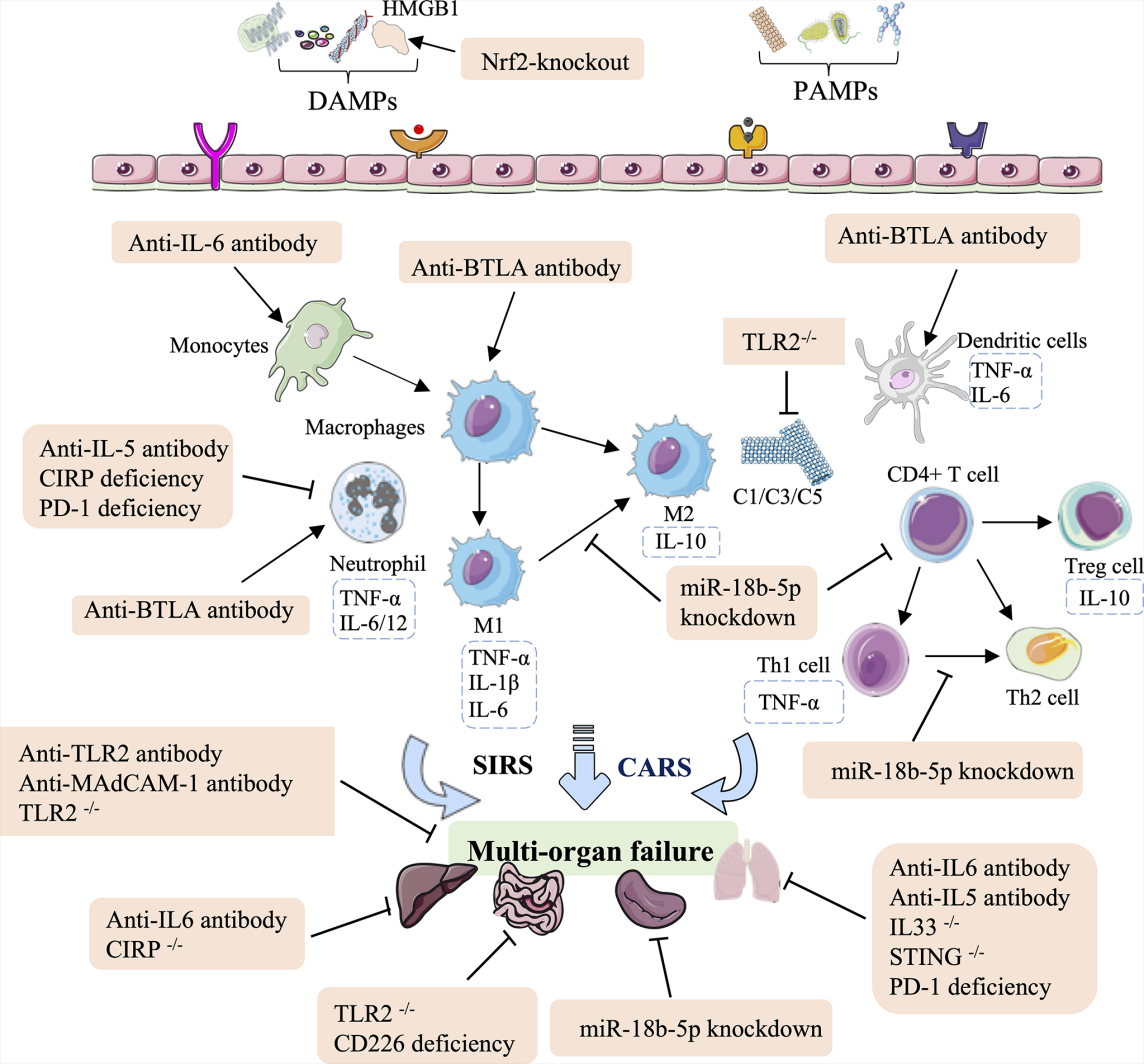
Overview of the immune therapeutic strategies of antibodies, and genetic approaches in hemorrhagic shock. The antibody of anti-IL-6, anti-TLR2, anti-IL-5, anti-IL-BTLA, and anti-MAdCAM-1; genetic approaches (RNAi-based deficiency such as PD-1, CD226, and miR-18b-5p); and gene knockout such as TLR2, IL33, CIRP, and STING can relieve lung, intestinal, splenic, liver, and multi-organ failure. These effects are mainly mediated by regulating the population of monocytes and macrophages, mediating neutrophil infiltration, reducing complement, the M1/M2 ratio in macrophages and the Th1/Th2 ratio in CD4^+^ T cells, and increasing recruitment of DCs.

Significant advances have been made in the identification of immune therapies for HS injury, including antibodies (anti-IL-6, anti-TLR2, anti-IL-5, anti-IL-BTLA, and anti-MAdCAM-1), RNAi-based deficiency (PD-1, CD226, and miR-18b-5p), and gene knockout (TLR2, IL33, CIRP, or STING). In summary, these antibody therapies and genetic approaches for HS are associated with a potent innate immune response that not only regulates the levels of inflammatory factors, the lymphocyte influx, and neutrophil infiltration, but also reduces complement, the ratio of M1/M2 macrophages, the Th1/Th2 ratio in CD4^+^ T cells, and increases recruitment of DCs (**Figure 3** and **Table 2**).

**Table 2:** Summary of the applications of antibody therapy and genetic approaches in the hemorrhagic shock model.

Treatment strategy	HS model	Mechanism	Inhibited outcome	Refs
Anti-IL-6 mAb	Mice	Preventing Th2 cytokine production, lymphocyte response, and the levels of IL-10, KDC, MCP-1, and MIP-1	Lung and liver injury	(67)
Anti-MAdCAM-1 mAb	Rat	Suppressing lymphocyte infiltration and the secretion of IL-1β, IL-6, and TNF-α	Mesenteric lymph injury	(70)
Anti-BTLA mAb	Mice	Increasing the levels of TNF-α, IL-12, IL-10, KC, and MIP-2, MCP-1, and promoting the recruitment of neutrophils, macrophages, and DCs	MOF injury	(75)
Anti-IL5 mAb or IL33^-/-^	Mice	Increasing IL-5 expression in neutrophil at 6 h	Lung injury	(77)
Anti-TLR2 mAb or TLR2^-/-^	Mice	Decreasing NF-κB and inflammatory cell infiltrates	MOF injury	(78)
TLR2^-/-^	Mice	Reducing complement (CD55, Factor H, and C3) and inflammatory factor (IL-12, IL-6 and TNF-α)	Intestinal injury	(79)
CIRP^-/-^	Mice	Reducing TNF-α, IL-6 secretion and neutrophil accumulation	Liver injury	(80, 81)
STING^-/-^	Mice	Decreasing the levels of IFN-β and IL-6 in the serum, and the mRNAs expression of TNF-α, and IL-1β and IL-6 in the lung	Lung injury	(84)
PD-1 deficiency	Mice	Suppressing neutrophil influx and release of TNF-α, MCP-1, and IL-6	Lung injury	(88)
CD226 deficiency	Mice	Inhibiting inflammation	Intestinal injury	(96)
miR-18b-5p knockdown	Rat	Reducing the Th1/Th2 ratio in CD4^+^ T cells in splenic tissues, the M1/M2 ratio in macrophages, and the levels of SOD1, iNOS, and IL-6 in macrophages	Spleen injury	(99)

### Small molecule inhibitor or agonist therapy in HS

As a master alarm system and a major fluid defense system of innate immunity after HS injury, the complement cascade can be rapidly activated by DAMPs or PAMPs, and lead to elevated plasma levels of complement activation products such as complement factor 1 (C1), complement factor 3 (C3) and complement factor 5 (C5) (100). As reviewed earlier, such exuberant complement activation evokes systemic inflammation, which is associated with increased susceptibility to infections and HS-induced MOF (8, 101). Early studies have shown that C3 deficiency attenuates HS-related hepatic injury and SIRS (102). The therapeutic inhibition of C3 by C3 inhibitor compstatin-40 (Cp40) is capable of improving immune, coagulation, and organ (kidney and intestine) functions by decreasing IL-6, MIF, IL-1RA, MIP-1, MCP-1, and IFN-γ (103). Another C3 inhibitor-soluble form of CR1 (sCR1) was confirmed to significantly mitigate the over-expression of NO, ET-1, TNF-α, and reactive oxygen species in serum to relieve vascular hyperreactivity in HS rats (104). Recombinant human C1-esterase inhibitor (rhC1-INH) has been found to particularly reduce tissue damage (kidney, gut, and lung), tissue complement activation, and cytokine release in an HS-induced porcine injury model (105). Additionally, the alternative complement activation in response to HS injury can induce macrophage infiltration and IL-12 secretion in the intestine (106). Multiple studies have indicated that treatment with complement inhibitors (C5 receptor antagonist or cobra venom factor) can significantly attenuate HS-induced intestinal injury (107–109). Furthermore, the mucosal damage, macrophage infiltration, and intestinal inflammation induced by HS injury were decreased by reducing leukotriene B4, IL-12p40, and TNF-α in the absence of IL-12p70 or treatment with complement receptor 2-targeted factor H (CR2-fH), a targeted inhibitor of the alternative complement pathway (106).

In addition to inhibitors targeting complement, many inhibitors targeting key proteins of innate immunity decrease HS-induced organ dysfunction. Recent studies have indicated that early intravenous treatment of tranexamic acid, a serine protease inhibitor, can protect the intestinal barrier by inhibiting neutrophil extracellular trap formation in the development of HS (110). CIRP acts as a DAMP to activate innate immunity and increases complications caused by HS (17). CIRP-derived oligopeptide-23 (C23) is homologous to the human CIRP protein (Ser110-Glu125) that binds to the CIRP receptor with high affinity and inhibits the secretion of TNF-α (111). Fangming Zhang et al. reported that the mRNA levels of IL-1β, TNF-α, and IL-6 in the lungs were reduced by adjuvant treatment with C23 (8 mg/kg) in HS-induced lung injury (111). Cyclosporine A (CsA) acts as a calcineurin inhibitor that participate in the innate immune response to pathogens in an inflammation storm (112). Some studies have shown that CsA could increase the survival time of HS rats by inhibiting proinflammatory cytokine production (IL-6) and reducing liver injury (113, 114).

Emerging evidence suggests that treatment with HDAC inhibitors (HDACIs) can attenuate MOF and improve early survival in animal models of HS by restoring “acetylation homeostasis” of histones and inducing transcriptional activation (115, 116). Transcriptomic studies in peripheral blood mononuclear cells (PBMC) and brain tissue suggested that valproic acid (VPA, one of HDACIs) can reduce HS-induced neurologic injury by downregulating genes associated with cell death and inflammation (IL-6, TLR4, JAK2, NLRP1, TNFα, IL-1α, IL-1B, NF-κB) (117, 118). In addition, treatment with VPA (150 mg/kg) significantly decreased brain lesion size and improved neurologic recovery by activating nuclear factor- k B (NF-κB) and degrading of cytosolic IκB in Yorkshire swine models of HS (119). Elizabeth A. Sailhamer et al. demonstrated that suberoylanilide hydroxamic acid (SAHA), one of HDACIs, can normalizes inflammatory cytokines (TNFα and IL-1β) levels by acetylating the transcription factor NF-κB following HS in the rats (120).

FTY720, an immunomodulator targeting receptors of sphingosine 1-phosphate (S1P), which can disrupt lymphocyte trafficking, prevent lymphocytes from accumulating in secondary lymphoid organs, and decrease lymphocytes in the blood circulation (121, 122). Jason S. Hawksworth et al. reported that FTY720 (0.3 mg/kg) could sequestrate the central lymphocytes, resulting in attenuation in innate cellular and signal activation following HS in a swine liver and lung injury model (123). FTY720 (1 mg/kg) has also been shown to reduce HS-induced MOD syndromes, red cell injury, and neutrophil priming in a rat model (124). The direct administration by a receptor agonist can block the binding of TLR ligands with their receptor, interfere with intracellular signaling molecules, and prevent signal amplification, which is a promising approach for treating HS-induced immune dysregulation. Xu Ding et al. reported that macrophage-activating lipopeptide-2 (MALP-2), as an agonist of TLR, given at the earliest can reduce pulmonary damage and polymorphonuclear neutrophil infiltration in an HS mouse model (125).

Latest studies have shown that some activators, such as sulforaphane, an Nrf2 pathway agonist, can modulate immunity against HS damage (126). Weiqiang Liang et al. demonstrated that sulforaphane, a potential immune modulator, could protect the liver from HS-induced inflammation storm by decreasing the secretion of TNF-α, MCP-1, KC/CXCL1, IL-6, and IL-10 and abolishing neutrophil infiltration in kupffer cells (126). Moreover, in a mouse HS model, sulforaphane administration reduced lung and liver injury *via* down-regulating pro-inflammatory cytokines, such as TNF-α, COX-2, iNOS, and IL-1β (127, 128).

**Figure 4:**
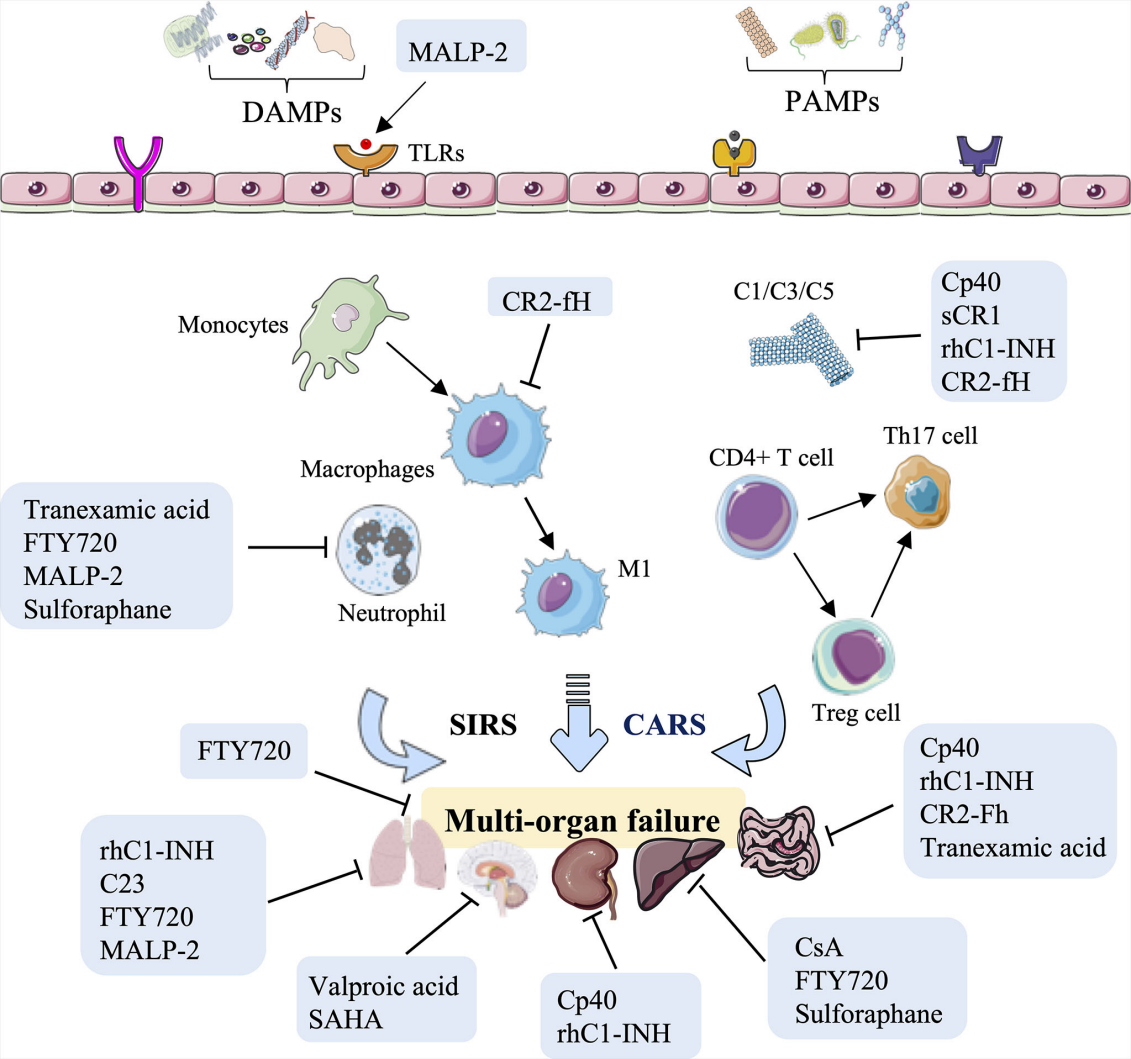
Overview of the immune therapeutic strategies of small molecule inhibitors or agonists in hemorrhagic shock. Small molecule inhibitors or agonists, especially complement-related target inhibitors, such as compstatin-40 (Cp40), soluble form of CR1 (sCR1), recombinant human C1-esterase inhibitor (rhC1-INH), complement receptor 2-targeted factor H (CR2-fH), tranexamic acid, CIRP-derived oligopeptide-23 (C23), FTY720, macrophage-activating lipopeptide-2 (MALP-2), cyclosporine A (CsA), and sulforaphane can reduce HS-induced liver, kidney, intestinal, lung, and multi-organ failure injury by reducing complement activation, macrophage infiltration, neutrophil priming, and systemic inflammation.

As discussed above, small molecule inhibitors, especially complement-related target inhibitors, inhibitors of serine protease, CIRP, sphingosine-1-phosphate, toll-like receptors, mPTP, and agonists of the Nrf2 pathway, can reduce HS-induced liver, kidney, intestinal, renal, and lung and vascular hyperreactivity injury in monkey, swine, and rodent animal models by modulating innate immune responses (**Figure 4**, **Table 3**).

**Table 3:** Summary of the applications of small molecule inhibitor or agonist therapy in a hemorrhagic shock model.

Name	Target	Conc.	HS model	Mechanism	Inhibitedoutcome	Refs
Cp40	Inhibitor of C3	3 mg/kg	Monkey	Reducing the levels of IL-6, MIF, MIP-1, MCP-1, and IFN-γ	MOF injury	(103)
sCR1	Inhibitor of C3	50 μg/kg	Rat	Reducing the levels of TNF-α and ET-1	Vascular injury	(104)
rhC1- INH	Inhibitor of C1	250 IU/kg	Porcine	Reducing TNF-α and complement levels	MOF injury	(105)
CR2-fH	Inhibitor of complement	17.5 µM	Mice	Reducing the levels of macrophages, IL-B4, IL-12p40, and TNF-α	Intestinal injury	(106)
Tranexamic acid	Inhibitor of serine protease	20 mg/kg	Rat	Inhibiting neutrophil extracellular trap formation	Intestinal injury	(110)
C23	Inhibitor of CIRP	8 mg/kg	Mice	Reducing IL-1β, TNF-α, and IL-6 levels	Lung injury	(111)
CsA	Inhibitor of mPTP	50 mg/kg	Rat	Decreasing the level of IL-6	Liver injury	(113, 114)
VPA	Inhibitor of histone deacetylase	150 mg/kg	Swine	Downregulating inflammatory pathways	Neurologic injury	(117–119)
SAHA	Inhibitor of histone deacetylase	400 nM	Rat	Normalizes TNFα and IL-1β	Improves survival	(120)
FTY720	Agonist of S1P	1 mg/kg	Rat	Inhibiting neutrophil priming	MOF injury	(124)
FTY720	Agonist of S1P	0.3 mg/kg	Swine	Increasing CD3^+^ T cell and inhibiting neutrophil priming	Liver and lung injury	(123)
MALP2	Agonist of TLRs	25 μg/kg	Mice	Reducing neutrophil infiltration	Lung injury	(125)
Sulforaphane	Agonist of Nrf2	50 mg/kg	Mice	Reducing the level of TNF-α, MCP-1, KC, IL-6, and neutrophils	Liver injury	(126)
Sulforaphane	Agonist of Nrf2	40 mg/kg	Rat	Decreasing the level of TNF-α and IL-1β	Liver injury	(127, 128)

### Natural medicine therapy for HS

Increasing research has confirmed the role of resveratrol, a natural polyphenol widely found in plants and fruits, in improving survival and prolonging lifespan following HS by improving immune function and reducing inflammation (129, 130). Phosphorylation and acetylation on the p65 subunit of NF-κB regulate the inflammatory cascade (131). In the HS-induced rat injury model, the ratio of the phosphorylated p65 subunit of NF-κB to the unphosphorylated form demonstrated a noticeable decline following resveratrol treatment (18). Resveratrol also counteracts the increase in gene expression and plasma secretion levels of IL-2, IL-6, IL-10, TNF-α, and MIP-1α at 2 h following HS in heart tissue (18). It has been reported that the release of proinflammatory cytokines caused by HS, participates in the development of kidney injury (132). Ophiopogonin A, an effective active component extracted from ophiopogonis radix, can dose-dependently downregulate the levels of iNOS, TNF-α, IL-1β and IL-6, and decrease HS-induced renal injury (133).

Intestinal DCs play essential roles in regulating the function of the intestinal immune barrier and intestinal bacterial translocation (134). Experimental evidence suggests that treatment with allicin, a thiosulfonate extract from freshly minced garlic, can block intraintestinal bacterial translocation and reduce the permeability of the intestinal barrier by assisting the immunologic barrier function of the ML node and facilitating the maturation of DCs (135, 136). Ursolic acid, a natural pentacyclic triterpenoid carboxylic acid isolated from uncaria rhynchophylla, reduces immune-mediated lung inflammation, assists human DCs *via* TLRs, and accelerates the production of IFNγ by CD4^+^ T cells (137, 138). Additionally, ursolic acid suppresses superoxide production in activated neutrophils and restrains HS-induced hepatic and lung injuries in rats (139). The development of complications from HS is accompanied by the activation of neutrophils (77, 140). In the rat HS model, the neutrophilic oxidative stress and lung injury were restrained after administration of osthol, a natural coumarin found in traditional medicinal plants (141).

**Figure 5:**
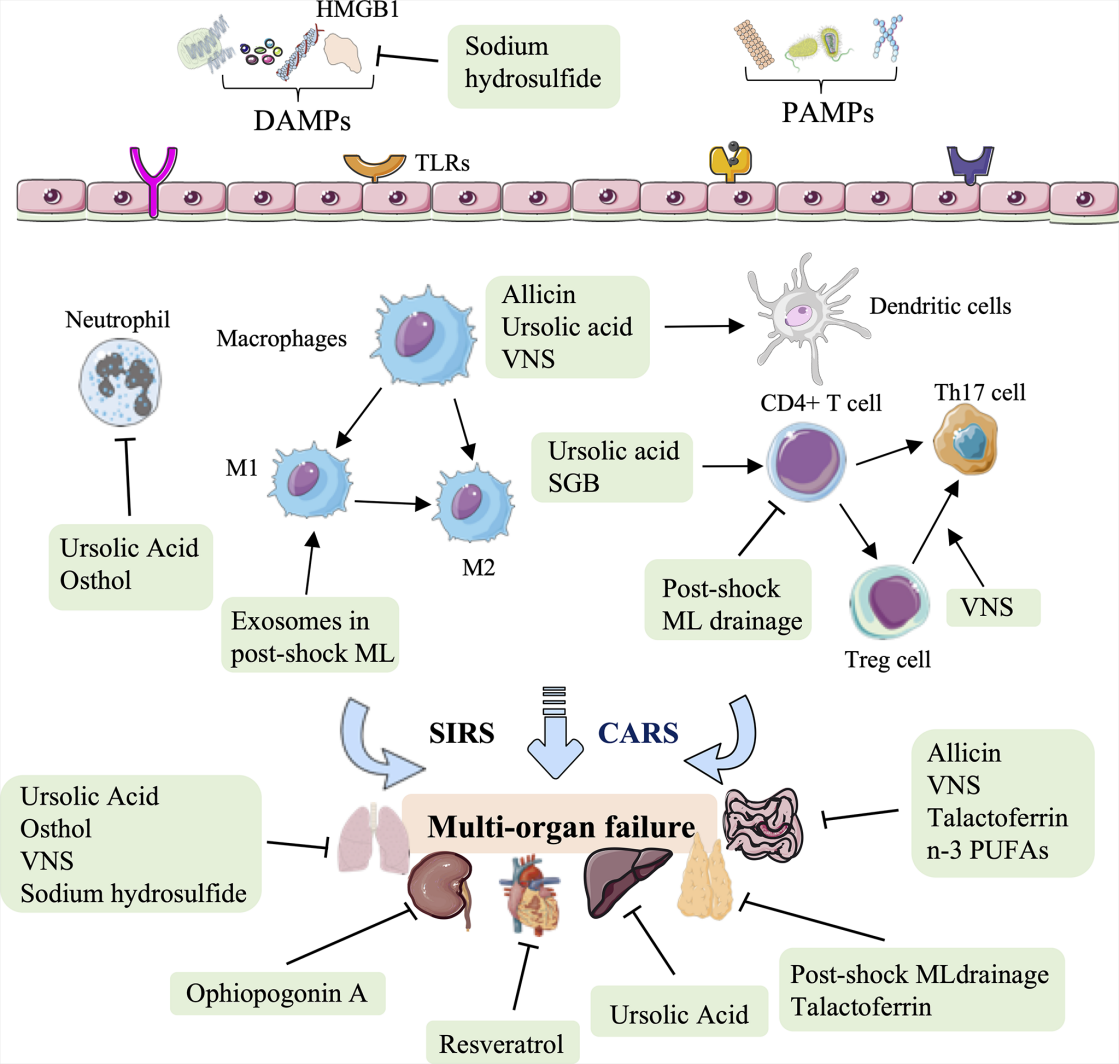
Overview of the natural medicine, vagus nerve stimulation, and other therapy approaches in hemorrhagic shock. Natural medicines, such as resveratrol, ophiopogonin A, allicin, ursolic acid, and osthol, act on several immune biological processes against HS-induced cardiac, liver, lung, and kidney injury, mainly rely on the inhibition of inflammatory cytokine release, modulating DC maturation and neutrophil-dominated inflammation. Other therapeutic approaches to HS, such as post-shock ML drainage, vagus nerve stimulation (VNS), stellate ganglion block (SGB), talactoferrin, n-3 polyunsaturated fatty acids (PUFAs), and hydrogen sulfide, restore the critical organs (thymus, lung, and gut) mainly by regulating the secretion of inflammatory and chemokines, and regulating the proportion of CD4 ^+^ T cell, Treg cells, Th17 cells, M1-type macrophages, and DCs.

In conclusion, natural medicines, such as polyphenol, saponins, thiosulfonate, carboxylic acid, and coumarin represent an essential therapy against HS-induced injury in terms of regulating immune processes, including subsiding inflammatory cytokine release, assisting the immunologic barrier function of the ML node, facilitating DC maturation, reducing superoxide production in activated neutrophils, and attenuating neutrophil-dominated inflammation, to improve cardiac function, block intraintestinal bacterial translocation, and relieve hepatic, lung, and kidney tissue injury (**Figure 5**, **Table 4**).

**Table 4:** Overview of the applications of natural medicine therapy in a hemorrhagic shock model.

Name	Compound type	Conc.	Mechanism	Inhibited outcome	Refs
Resveratrol	Polyphenol	10 mg/kg	Inhibiting the levels of NF-κb(p65), IL-6, TNF-α, IL-2, MIP-1α, and IL-10	Cardiac injury	(18)
Ophiopogo-nin A	Saponins	Not mentioned	Reducing the levels of TNF-α, IL-1β, and IL-6	Kidney injury	(133)
Allicin	Thiosulfo- nate	Not mentioned	Increasing DC maturation	Intestinal damage	(135)
Ursolic Acid	Carboxylic acid	1 mg/kg	Inhibiting superoxide production in neutrophils	MOF	(139)
Osthol	Coumarin	1 Um	Attenuating neutrophil-dominated inflammatory	Lung injury	(141)

### Other therapies in HS

Other therapies, such as physical therapy (post-shock ML drainage, VNS, stellate ganglion block), glycoprotein, fatty acids, and inorganic compounds can also be used to modulate immunity against HS injury (**Figure 5**, **Table 5**). Previous studies have shown that the diversion of the ML or lymphatic duct ligation can reduce vascular permeability, subside systemic neutrophil priming, and decrease lung injury in HS models (143). It has been recently shown that post-shock ML drainage can decrease the levels of the T lymphocyte subgroup, including the population of CD3^+^ cells, CD3^+^ cells, CD4^+^ cells, and CD4^+^CD25^+^ cells, and reduce IFN-γ and IL-4 secretion in the HS rat model at 3 h after resuscitation, which suggests that post-shock ML drainage can markedly improve hyperimmunity occurred at early stages (142). Conversely, exosomes isolated from post-shock ML significantly increase lung injury by recruitment of inflammatory cells to the alveolar space and lung parenchyma, inducing mRNA expression of NF-κB, iNOS, TNF-α, and CINC-1 during HS (150).

**Table 5:** Summary of the applications of physical therapy, glycoprotein, fatty acids, and inorganic compounds in the hemorrhagic shock model.

Treatment strategy	Type of therapy	HS model	Mechanism	Inhibited outcome	Refs
Post-shock ML drainage	Physical therapy	Rat	Restoring the T lymphocyte subgroup and IFN-γ/IL-4 ratio	Thymus injury	(142)
VNS	Physical therapy	Rat	Increasing the DC population and the ratio of Treg/Th17	Intestinal injury	(143)
VNS	Physical therapy	Rat	Increasing the IL-10 level, and decreasing the levels of TNF-α, IL-6, NF-κB, and MPO	MOF injury	(144, 145)
Stellate ganglion block (SGB)	Physical therapy	Rat	Increasing CD4 ^+^ T cell population and the levels of IL-2, IL-4, and TIPE	Mesenteric lymph return	(146)
Talactoferrin	Glycoprotein	Rat	Reducing the biological activity of lymph	Intestinal injury	(147)
n-3 PUFAs	Fatty acids	Mice	Balancing the levels of IL-10 and IL-4	Intestinal injury	(148)
Sodium hydrosulfide	Inorganic compounds	Rat	Decreasing the release of IL-6, TNF-α, and HMGB1	Lung injury	(149)

The continuous migration of CD103^+^ DCs from the intestine to the ML nodes is considered to induce Treg cell maturation and promote tolerance to intestinal inflammation (151, 152). The balance of Treg and Th17 cells determines the intestinal tolerance to inflammation and immune response (153). Previous studies have demonstrated that VNS prevents HS-induced impairment in intestinal blood flow, alters the DC profile, and prevents incompleteness of the gut barrier in the ML (154–158). Additionally, Koji Morishita et al. reported that VNS could promote tolerance to HS-induced inflammation by increasing the CD103^+^ DC population in the ML and facilitating the ratio of Treg cells to Th17 cells (143). VNS has also been shown to increase the level of IL-10 and decrease HS-induced lung and gut barrier injury, with a marked decrease in the mRNA level of TNF-α, IL-6, NF-κB, and MPO (144, 145). Previous studies have shown that stellate ganglion block (SGB), a standard method of blocking sympathetic nerves, can reduce intestinal barrier dysfunction and prolong the survival time in the HS rat model (159). The latest research shows that SGB administration significantly normalized the population of CD4^+^ T cells and the level of IL-2, IL-4, and TNFα-induced protein 8 like 2 (TIPE2) in the development of HS (146).

Lactoferrin, as a pleiotropic glycoprotein, was proven to limit HS-induced gut injury and inhibit the biological activity of ML by enhancing the gastrointestinal barrier and assisting mucosal immunity (147). Talactoferrin, a unique recombinant form of human lactoferrin and an oral DC cell-mediated immunotherapy, has demonstrated safety and preliminary efficacy in clinical trials (160–162). It has been recently shown that talactoferrin (1000 mg/kg/day) pretreatment 5 d before being subjected to HS injury has gut-protective effects by reducing the respiratory burst activity of lymph (147). Intestinal mucosa innate immunity is involved in removing pathogenic bacteria and alleviating intestinal injury (163). Feng Tian et al. reported that n-3 polyunsaturated fatty acids (PUFAs) could restore the function of the intestinal barrier by improving the innate immunity of the intestinal mucosa, increasing the expression of lysozyme, mucin 2, and IL-4, and stabilizing the intestinal microbiota in mice after HS (148). An early study revealed that hydrogen sulfide could increase survival in rodent models of lethal hemorrhage (164). Moreover, Dunquan Xu et al. demonstrated that sodium hydrosulfide administration could protect lungs against HS injury by suppressing the levels of IL-6, TNF-α, and HMGB1 in rat bronchoalveolar lavage fluid (149).

In summary, increasing therapeutic approaches to HS have been verified, focusing on various mechanisms involving innate immunity. These therapeutic approaches restore the thymus, lung, and gut. In addition to regulating the secretion of inflammatory cytokines and chemokines (IFN-γ, IL-4, NF-κB, iNOS, TNF-α, IL-10, IL-6, IL-2, and CINC-1), these novel therapeutic options for treating HS to restore critical organ function mainly rely on regulating the population of innate immune cells, involving T lymphocyte subgroup, CD4 ^+^ T cell, Treg cells, monocytes, Th17 cells and DC cells (**Figure 5**, **Table 5**).

### Clinical opportunities of innate immune modulation in HS

Although some attempts at targeting innate immunity against HS injury and subsequent organ damage in preclinical models have been successful, only a few clinical trials have evaluated the treatment strategies with immune-related indicators as primary or second outcome measures in patients with HS.

Dexmedetomidine (Dex; α2 adrenergic receptor agonist) targeted activation of α2 receptors can produce sedative, analgesic, antisympathetic, and hemodynamic effects (165). New clinical evidence indicates that early intervention with Dex can effectively prevent postoperative renal insufficiency or renal failure and improve microcirculation in patients with HS requiring surgery, mainly by inhibiting the release of oxygen free radicals, IL-6, and IL-8 (166). Another promising therapeutic compound is ulinastatin, a glycoprotein derived from human urine; combined with thymosin α1, ulinastatin improves the survival rate of patients with bacterial infection by significantly increasing the CD4^+^CD8^+^ population and restoring the balance between proinflammatory mediators (TNFα, IL-1β, IL-6, and IL-8) and anti-inflammatory cytokines (IL-4 and IL-10) (167). Park et al. reported that ulinastatin administration (300,000 IU) neutralizes the serum polymorphonuclear leukocyte elastase (PMNE) levels and decreases the secretion of TNF-α and IL-6 in trauma patients with HS at 48 h after administration (168). Pre-clinical studies have certificated the benefit of estrogen in reducing MOF injury and mortality in HS (169, 170). More importantly, the levels of Treg cell, monocytes, and inflammatory factors were significantly balanced in patients with HS after receiving estrogen treatment (171). Conversely, the results of one clinical study on the effect of remote ischemic conditioning on trauma patients with HS using immune regulation (neutrophil activity and plasma inflammatory factor expression) as the primary outcome measures were disappointing, limited by prolonged emergency transport time and delayed application of therapy (ClinicalTrials.gov Identifier: NCT02071290). One therapeutic method for HS injury currently under clinical investigation is hypertonic resuscitation, with the primary outcome measures being neutrophil activation, coagulation parameters, and monocyte activation (ClinicalTrials.gov Identifier: NCT00750997).

Most of the clinical studies of HS therapy have focused on resuscitation fluid and modulators (nitroglycerine, polydatin, vasopressin, estrogen, and the combination of norepinephrine with octreotide) using hospital admission rate, urinary output, blood pressure, heart rate, Glasgow coma scale value, microcirculatory flow index, perfusion index, mean arterial pressure, survival, or organ dysfunction as the primary outcome measures (ClinicalTrials.gov Identifier: NCT01780129, NCT03891849, NCT03235921, NCT01433276, NCT00379522, NCT00973102) (172–175). There remains an immense need to validate these promising strategies targeting innate immunity against HS injury in non-human primate models, organoid models, and clinical patients.

## Conclusion and prospects

Increasing experimental and clinical evidence has contributed to a profound understanding of the pathophysiology of HS injuries in recent years. Furthermore, regulation of innate immunity is recognized as an attractive pharmacological target offering encouraging future directions for the R&D of novel therapeutics. In this regard, numerous strategies, including MSCs, exosomes, genetic approaches, antibody therapy, small molecule inhibitors, and natural medicine, have been successfully employed for protection from HS damage and MOF in rodents, porcine, and non-human primate models. The immunomodulatory mechanisms of therapeutic approaches in HS injury discussed above are not only reflected in the regulation of inflammatory cytokines and chemokines but also in the balance of complement, DCs, macrophages polarization, T lymphocyte differentiation, and neutrophil infiltration.

The systematic and comprehensive research focus on HS injury and innate immunity regulation has led to many advances; however, gaps in the translation from basic research to clinic capability remain. As immune activation and immunosuppression are inseparable and sequential during the development of MOF in HS, the time point of starting and stopping therapeutic interventions is crucial. Furthermore, reliable monitoring of the remaining immunomodulatory functions of the intervening strategies within the HS-induced immunity response cascade are equally important. Ongoing studies should accelerate the progression of the most promising strategy targeting innate immunity to clinical trials in HS injury. Overall, the modulation of the innate immune response by specific intervening strategies might provide the key to closing the cascading damage resulting from the vicious danger response after HS injury.

## Author contributions

QH, DZ, and XL conceived and designed the review. QH wrote the first draft of the manuscript. DZ and XL wrote sections of the manuscript. SG and YY conceived and drafted the figures. YW and JL guided part of the manuscript. JC and CG critically revised the final manuscript. All authors contributed to the article and approved the submitted version.

## Funding

This work was supported by the National Natural Science Foundation of China (Grant No. 82104432 and U19A2013), and the Science and Technology Development Plan Project of Jilin Province (Grant No. 202002053JC and 20200201419JC).

## Conflict of interest

Author SG is employed by JX Pharmaceutical New Drug Development Co., Ltd.

The remaining authors declare that the research was conducted in the absence of any commercial or financial relationships that could be construed as a potential conflict of interest.

## Publisher’s note

All claims expressed in this article are solely those of the authors and do not necessarily represent those of their affiliated organizations, or those of the publisher, the editors and the reviewers. Any product that may be evaluated in this article, or claim that may be made by its manufacturer, is not guaranteed or endorsed by the publisher.
